# Cost-Effectiveness of Pneumococcal Vaccination in Adults in Italy: Comparing New Alternatives and Exploring the Role of GMT Ratios in Informing Vaccine Effectiveness

**DOI:** 10.3390/vaccines11071253

**Published:** 2023-07-18

**Authors:** Vincenzo Restivo, Vincenzo Baldo, Laura Sticchi, Francesca Senese, Gian Marco Prandi, Linde Pronk, Kwame Owusu-Edusei, Kelly D. Johnson, Tim Ignacio

**Affiliations:** 1 Department of Health Promotion, Mother-Child Care, Internal Medicine and Medical Specialties, University of Palermo, 90127 Palermo, Italy; 2 Hygiene and Public Health Unit, Department of Cardiac, Thoracic, Vascular Sciences and Public Health, University of Padua, 35131 Padua, Italy; 3 Department of Health Sciences, University of Genoa, 16100 Genoa, Italy; 4 Market Access, MSD Italy, 00189 Rome, Italy; 5 Medical Affairs, MSD Italy, 00189 Rome, Italy; 6 OPEN Health Group, 3068 AV Rotterdam, The Netherlands; lindepronk@openhealthgroup.com (L.P.);; 7 Merck & Co., Inc., Rahway, NJ 07065, USA

**Keywords:** cost-effectiveness models, pneumococcal disease, sequential vaccination, Italy, non-bacteremic pneumococcal pneumonia, geometric mean titer

## Abstract

In Italy, a sequential pneumococcal vaccination with conjugate vaccine (PCV) and polysaccharide vaccine (PPSV23) is recommended for individuals aged ≥ 65 years and those at risk for pneumococcal disease (PD) aged ≥ 6 years. The aim of this study was to assess the cost-effectiveness of the new vaccines, i.e., approved 15-valent and 20-valent PCVs. A published Markov model was adapted to evaluate the lifetime cost-effectiveness of vaccination with PCV15 + PPSV23 versus PCV13 + PPSV23, PCV20 alone, PCV20 + PPSV23, and No Vaccination. Simulated cohorts representing the Italian population, including individuals aged ≥ 65 years, those at risk aged 50–100 years, and those deemed high risk aged 18–100 years were assessed. Outcomes were accrued in terms of incremental PD cases, costs, quality-adjusted life years, life years, and the cost–utility ratio relative to PCV13 + PPSV23. The conservative base case analysis, including vaccine efficacy based on PCV13 data, showed that sequential vaccination with PCV15 or PCV20 in combination with PPSV23 is preferred over sequential vaccination with PCV13 + PPSV23. Especially in the high-risk group, PCV15 + PPSV23 sequential vaccination was dominant over No Vaccination and resulted in an ICUR of €3605 per QALY gained. Including PCV20 + PPSV23 into the comparison resulted in the domination of the PCV15 + PPSV23 and No Vaccination strategies. Additionally, explorative analysis, including the geometric mean titer (GMT) informed vaccine effectiveness (VE) was performed. In the low-risk and high-risk groups, the results of the GMT scenarios showed PCV15 + PPSV23 to be dominant over the other sequential vaccines. These findings suggest that if real-world studies would confirm a difference in vaccine effectiveness of PCV15 and PCV20 versus PCV13 based on GMT ratios, PCV15 + PPSV23 could prove a highly immunogenic and effective vaccination regime for the Italian adult population.

## 1. Introduction

### 1.1. Pneumococcal Epidemiology

Pneumococcal disease (PD) is caused by *S. pneumoniae* and is divided into non-bacteremic pneumococcal pneumonia (NBPP) and invasive pneumococcal disease (IPD) [1]. Together, NBPP and IPD are a major cause of adult morbidity and mortality globally [2]. Among the Italian elderly (aged 65+ years), the latest estimated annual IPD incidence is 7.26 per 100,000 people per year (1703 cases) as of 2019, with fatality rates increasing by age [3,4]. Over the course of 2010 to 2017, Italy has seen an increase in IPD cases among the elderly [5]. A similar pattern has been observed for pneumonia-associated hospitalization rates that, according to several Italian studies, peak among adults aged over 65 years, with an estimated proportion of 37.2% attributed to *S. pneumoniae* [6,7]. As vaccination is the sole intervention shown effective in reducing PD, the implementation of an effective vaccine covering the most prevalent PD serotypes is crucial to limit the increase in IPD cases and hospitalization rates [8,9].

### 1.2. Pneumococcal Vaccination in Italy

The 23-valent pneumococcal polysaccharide vaccine (PPSV23) and the 13-valent pneumococcal conjugate vaccine (PCV13) are currently the recommended vaccines for older Italian adults by the National Immunization Plan (in Italian: Piano Nazionale di Prevenzione Vaccinale; NIP), where the use of sequential regimens of a PCV followed by PPSV23 is the official recommendation both for adults 65 or older without risk conditions and for immunocompromised individuals [10]. The European Medicines Agency has recently approved the use of a new 15-valent conjugate vaccine (PCV15), which is specifically of interest due to its inclusion of the 22F and 33F serotypes that jointly account for 7.7% of IPD cases in Italian adults 65 or older [11]. The other most common IPD-causing serotypes in Italy are serotypes 3 (19%) and 8 (21%), of which the latter is included in the 20-valent conjugate vaccine (PCV20), covering the PCV13 serotypes plus 8, 10A, 11A, 12F, 15BC, 22F, and 33F [12], and PPSV23 [3]. 

### 1.3. Objective

Policymakers face increasing complexity in their PD vaccination selection process due to the introduction of new PCVs into the market. The lack of head-to-head comparisons for these vaccines creates a research gap and poses a challenge for policymakers in assessing their relative cost-effectiveness compared to existing vaccination schemes. To bridge this knowledge gap, our study investigates the comparative cost-effectiveness of vaccinating Italian adults with PCV15 + PPSV23, PCV13 + PPSV23, PCV20 + PPSV23, PCV20 alone, or No Vaccination. The findings from this research provide essential information to inform decision making in public health policy.

Furthermore, exploratory analysis was performed for the use of geometric mean titers (GMT) ratios, as serotype-specific immune responses are gaining recognition as potential predictors of protection in clinical practice [13]. By leveraging GMT ratios, we derive alternative efficacy estimates for PCV15 and PCV20 compared with PCV13.

## 2. Materials and Methods

### 2.1. Model Structure

**Figure 1:**
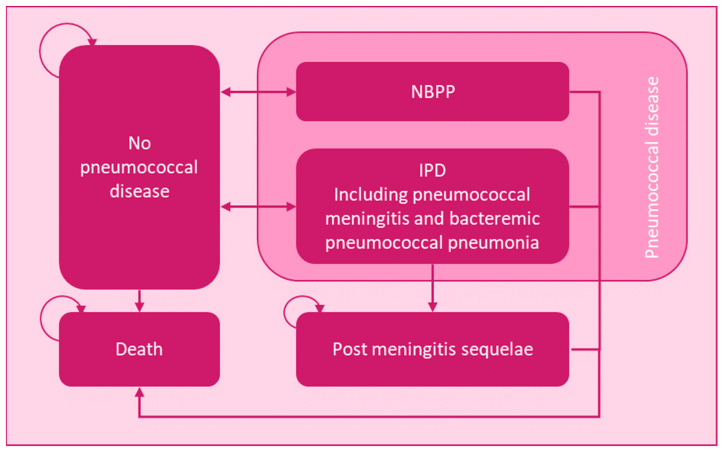
Markov model structure. Abbreviations: IPD = invasive pneumococcal disease; NBPP = non-bacteremic pneumococcal pneumonia. Background mortality is applied to all health states in the model.

To assess the cost-effectiveness of PCV15 + PPSV23 versus other strategies in various adult risk groups in Italy, a previous published multi-state Markov model was used (Figure 1). The complete details of the Markov model structure are described in Deb et al., 2022 [14].

The Italian model originated from the published model by populating the published model with Italy-specific inputs and aligning model functionality settings with the Italian market for PD in the adult population. Key functionalities adopted for the Italian market were serotype-specific efficacy and efficacy waning, which are further elaborated on in Section 2.3.

The health states included are No PD, NBPP, IPD, post-meningitis sequelae (PMS), and death. Patients enter the model PD free and can subsequently develop either NBPP or IPD. Each 1-year cycle, patients can either recover from their disease and return to the no PD state, remain ill and hence stay in their NBPP or IPD state, develop PMS following IPD meningitis, or die. Death itself is an absorbing state. Costs and outcomes were discounted at 3% [15] and accrued over a time horizon based on the youngest cohort, such that a life expectancy of 100 years was accounted for. Half-cycle correction was applied to avoid skewed outcome aggregation. 

### 2.2. Population, Intervention, and Comparator

In the Italian setting, three risk groups eligible for pneumococcal vaccination are stratified. The low-risk group includes healthy adults aged 65 years old who are eligible for vaccination, as recommended by the NIP. This risk group comprises approximately 53.18% of the 65-year-old cohort (*N* = 387,993), according to the National Survey on Health Status (Multiscopo) [16,17]. The at-risk group includes individuals aged 50 to 100 years who have one or more underlying conditions or risk factors that make them more susceptible to developing PD (e.g., chronic heart disease, chronic respiratory disease, liver disease, or diabetes mellitus). Collectively, 37.75% of adults aged 50+ years are considered at risk (*N* = 10,255,405). Finally, the high-risk immunosuppressed group accounts for 9.07% of individuals aged 18 to 100 years (*N* = 4,552,553), who suffer from underlying morbidities compromising their immune system [18].

The intervention of interest for all risk groups is the new PCV15 vaccine in combination with the currently recommended PPSV23 vaccine. The currently recommended sequential regimen of PCV13 + PPSV23 and No Vaccination are both considered as comparators for all risk groups. PCV20, both by itself and in combination with PPSV23, is not included as a comparator for the high-risk group due to a lack of clinical studies investigating PCV20 in immunocompromised patients [19].

### 2.3. Clinical Inputs

Clinical inputs include baseline incidence and mortality of IPD and NBPP, NBPP-related hospitalization, and vaccine efficacy (VE) and coverage. These allow for calculation of the number of PD cases and deaths as well as further quantification of costs and outcomes in each of the vaccination strategies.

Patients with PD are subject to a higher fatality risk compared with healthy individuals. Additionally, nationally reported life tables are used to account for background mortality [16]. Baseline PD incidence and mortality increase with age and are dependent on risk group allocation (Supplementary Material, Tables S1–S3). Serotype-specific baseline IPD incidence is based on official data from 2019 reported by the Italian surveillance system (MaBI) Report [3]. Incidence rates are corrected by applying European Surveillance (TESSy) incidence rates to account for the MaBI underreporting [20].

A reduction in baseline PD incidence and mortality is achieved through a vaccine’s efficacy, which applies to the serotypes covered by each vaccine and is assumed to wane linearly over time. All non-vaccine type (NVT) serotypes are not affected and are assigned an efficacy of 0% in an aggregated NVT bucket. VEs used in this analysis are depicted in Table 1 and are derived from recent cost-effectiveness analyses that further separated VE for IPD and NBPP. VE for all modeled vaccines was based on the published PCV13 VE, and instead of having individual vaccine efficacy for every serotype separately, only VE was divided into serotype 3 VE and all others for PCV vaccines and a single efficacy for serotypes within PPSV23 vaccine. To account for differences in resistance to antibiotics between serotypes and their consequent effect on PD incidence, we include serotype-specific probabilities of antimicrobial resistance (AMR) in IPD and NBPP (Table 2).

As VE decreases over time, the model simulates vaccine waning functions. Based on the most adopted waning pattern, indicated by Treskova et al., 2019 [28], we assume PCVs to maintain their efficacy for the first five years after vaccination, which is followed by a linear decline that reaches 0% efficacy at 20 years. As PPSV23’s efficacy deteriorates faster, we assume that the linear decline starts after two years and reaches 0% efficacy at 15 years. For sequential regimens, waning starts after PCV administration and is reset to maximum efficacy for the serotypes covered in PPSV23 after sequential vaccination with PPSV23. Subsequently, PCV serotypes continue their original waning pattern, while the PPSV23 serotypes follow the PPSV23-specific waning pattern.

Due to the lack of real-world studies on new PCVs, in the base-case scenario, new PCVs are assumed to have equal VE to PCV13 across shared serotypes. However, applying PCV13’s observed VE to the shared serotypes also covered by PCV15 and PCV20 limits the evaluation of differential costs and benefits associated with PCV15 and PCV20 merely to their additional serotype coverage.

Therefore, the opsonophagocytic activity (OPA) GMT [29] ratios derived by Mt-Isa et al. were used as an explorative proxy of the new vaccines’ efficacy [13]. The methodology proposed by Mt-Isa et al. comprises an indirect comparison of GMT ratio estimates for PCV15 and PCV20 versus PCV13, which were applied to the base case VE of PCV13-specific serotypes [13]. The effects of these estimates were investigated in separate scenario analyses. 

The vaccine coverage rate (VCR) affects PD incidence and consequently PD mortality in the model. Even though adult pneumococcal VCRs are not routinely recorded in Italy, based on our analysis of doses sold for PCV13 and PPSV23 in the period 2017–2020, along with expert validation, we estimate that the VCRs are highest in the low-risk group (65% at the age of 65 when local health units offer this immunization), and we assume 50% in the high-risk immunosuppressed group and 25% in the at-risk group [30].

### 2.4. Utilities

Each risk group is assigned its own baseline utilities, which decrease as patients become older, to reflect risk group–associated quality of life (Supplementary Material, Table S6). We assume quality of life to be equal and decreasing with age, across at-risk and high-risk patients, and for patients with low risk to have higher quality of life at all ages compared with the other two risk groups. Utility decrements are included to reflect the decrease in quality of life due to IPD and NBPP and are multiplied by the number of days spent in the respective health state (Supplementary Material, Table S7) to assess the effect on quality-adjusted life years (QALYs) [30].

### 2.5. Cost Inputs

Costs in the base case setting are considered from a societal perspective and include vaccine-specific costs, medical costs, and indirect costs. Vaccine acquisition costs (Table 3) come from publicly reported prices [31], which already account for the mandatory 50% discount to the Italian National Health Service according to Italian law [32]. As market prices for PCV15 and PCV20 were not yet known at the time of analysis, PCV15 and PCV20 are assumed to be equally priced to PCV13.

Medical costs and indirect costs are specified for IPD, meningitis, PMS, NBPP inpatient and outpatient visits, and AMR costs for IPD and NBPP (Table 4). The latter accounts for incurred costs of hospitalization and death due to the antimicrobial resistance of *S. pneumoniae* to most antibiotics. Indirect costs related to PMS are based on Ansaldi et al. [34] and increase with age. Prices were not inflated using the Consumer Price Index due to the variety of the sourced costs and their publication year, although most are based on the latest official NHS tariff system published in 2013, which specified costs of hospitalization (diagnostic-related groups) and outpatient visits [33].

### 2.6. Outcomes

For each risk group, discounted outcomes are reported as the number of PD events, direct and indirect costs, quality-of-life outcomes, and incremental outcomes. The number of PD events is further separated out into new cases of IPD (meningitis + bacteremia and meningitis alone), NBPP (inpatient and outpatient), PMS, and AMR in IPD and NBPP (inpatient and outpatient). Costs are reported for vaccination, administration, IPD, NBPP, meningitis, PMS, and AMR. Health outcomes are measured in terms of QALYs and life years. Incremental results are presented for costs, QALYs, life years and cost-effectiveness, which is expressed in terms of the incremental cost–utility ratio (ICUR).

The main drivers of model outcomes and parameter uncertainty effects on model outcomes are analyzed in deterministic (DSAs) and probabilistic sensitivity analyses (PSAs). DSA results are displayed by tornado diagrams with ICURs reported for the upper and lower limits of the included parameters, while PSA results are displayed by scatter plots of the cost-effectiveness plane and the cost-effectiveness acceptability curve (CEAC).

Eight additional scenario analyses are conducted for each of the risk groups to quantify the effects of input assumptions on the ICUR. Variation of the vaccine coverage rates is tested by increasing the coverage rate for the low-risk group from 65% to 75%, the vaccination target recommended in the NIP, and for the at-risk and high-risk groups from 25% and 45%, respectively, to 100%, acknowledging that vaccinating individuals at higher risk is particularly beneficial. Additionally, the acquisition price for PCV15 is discounted at 5% compared to the price for PCV13. As IPD incidence rates are corrected in the base case analysis using TESSy rates, a scenario tests the impact of using the IPD cases’ incidence as reported by the MaBI, which are halved compared with the base case. The impact of NBPP incidence is tested by halving NBPP incidence rates as well. In the base case analysis, vaccine efficacy waning is incorporated by assuming a constant efficacy for a specified period, which is followed by a linear decline. This approach aligns with the commonly assumed waning pattern in the literature. Additionally, we explore two additional scenarios that consider other observed waning patterns. Since empirical data informing the efficacy waning of each individual PCV are currently lacking, these scenarios provide valuable insights into the potential impact of different waning assumptions. Furthermore, the estimated comparative vaccine effectiveness of PCV15 and PCV20 versus PCV13 is implemented in a scenario to analyze the effects of GMT ratios. The derived GMT VE inputs for PCV15 and PCV20 are displayed in the Supplementary Material; see Tables S4 and S5. The final two scenarios combine the derived GMT VE inputs with the discounted acquisition price for PCV15 and the GMT VE with increased coverage rates for each of the risk groups.

## 3. Results

### 3.1. Base Case Results

Table 5, Table 6, Table 7 and Table 8 report the number of new PD cases, the costs associated with the various vaccination strategies, and the incremental outcomes for all three risk groups. With coverage rates varying between 25% and 65%, the number of new IPD and NBPP cases, deaths, and hospitalizations drop in all risk groups following vaccination. As the baseline NBPP incidence is relatively high in Italy, the largest gain is seen in NBPP cases and related hospitalization and fatalities. 

When comparing PCV15 + PPSV23 versus the currently recommended vaccination strategy, PCV13 + PPSV23, the number of new IPD and NBPP cases, deaths, hospitalizations, and the associated costs are lower for PCV15 + PPSV23 in all risk groups, while an increase in QALYs and life years is seen. As such, in all risk groups, PCV15 + PPSV23 dominates PCV13 + PPSV23. Comparing the two newly approved vaccination strategies of PCV15 + PPSV23 and PCV20 + PPSV23, the QALYs and life years gained are higher for the PCV20 + PPSV23 strategy, leading to PCV20 + PPSV23 dominating PCV15 + PPSV23 in both low-risk and at-risk individuals. PCV20 as a single vaccination dominates PCV15 + PPSV23 in the low-risk and at-risk groups as well. When vaccinating with PCV15 + PPSV23 compared to No Vaccination, total costs to society increase in the low-risk and at-risk groups, which are mainly driven by vaccine acquisition and administration costs. Consequently, the ICURs of PCV15 + PPSV23 versus No Vaccination are €108,542 and €3605 per QALY gained among the low-risk and at-risk groups, respectively. Vaccination with PCV15 + PPSV23 dominates No Vaccination in high-risk patients.

### 3.2. Deterministic Sensitivity Analyses

**Figure 2:**
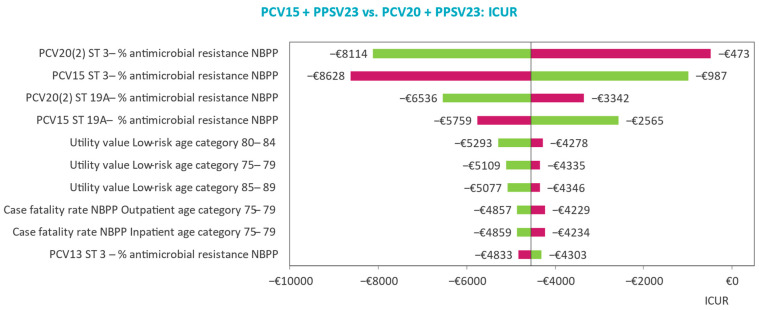
Tornado plot identifying top 10 drivers of the model’s incremental outcomes for the low-risk group.

**Figure 3:**
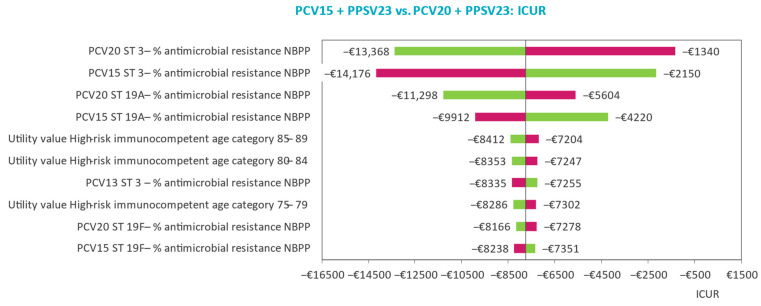
Tornado plot identifying top 10 drivers of the model’s incremental outcomes for the at-risk group.

**Figure 4:**
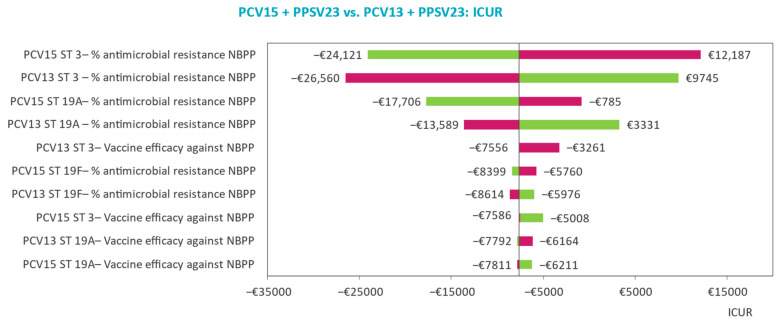
Tornado plot identifying top 10 drivers of the model’s incremental outcomes for the high-risk group.

The main drivers of the model outcomes are identified in the DSA. In all three risk groups, the top 10 most influential parameters largely concern the probability of AMR for NBPP and age-specific utility and serotype-specific vaccine efficacy in NBPP (Figure 2, Figure 3 and Figure 4). The influence of varying the probability of AMR for NBPP leads to most variability in the ICUR. Despite this, sequential vaccination with PCV20 always dominates sequential vaccination with PCV15 when varying serotype 3- and 19-specific AMR in NBPP in patients at low risk and ‘at-risk’.

In the comparison of PCV15 + PPSV23 versus PCV13 + PPSV23 for the high-risk group, domination is maintained in the DSA, except when serotype 3 AMR in NBPP is set to the lower limit, leading to an ICUR of €12,187 per QALY gained versus PCV13 + PPSV23.

### 3.3. Probabilistic Sensitivity Analyses

Scatter plots of incremental costs and QALYs resulting from 1000 PSA iterations show results that largely align with the base case results (Supplementary Material, Figures S1–S3). For the at-risk and high-risk group, there is little variance in incremental QALYs but more in incremental costs (Supplementary Material, Figure S2 and Figure S3, respectively), which suggests base case results in these groups come with some uncertainty. However, probabilistic ICURs do align with the base case, and the scatter plots support the findings of the base case, where PCV15 + PPSV23 dominates both the current strategy of PCV13 + PPSV23 as well as No Vaccination.

**Figure 5:**
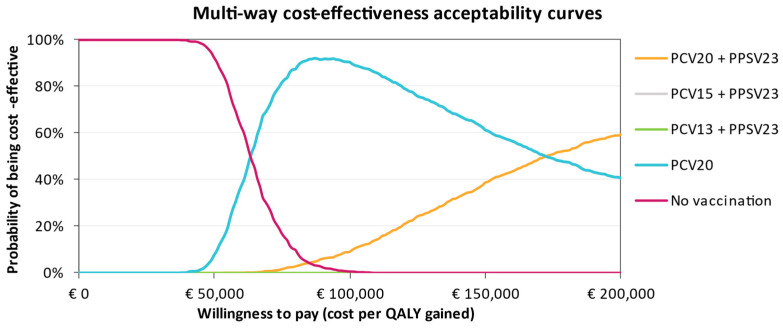
Cost-effectiveness acceptability curve of all vaccination strategies for the low-risk group. Where comparators are seemingly not visible in the CEACs, their probability of being cost-effective is 0%.

**Figure 6:**
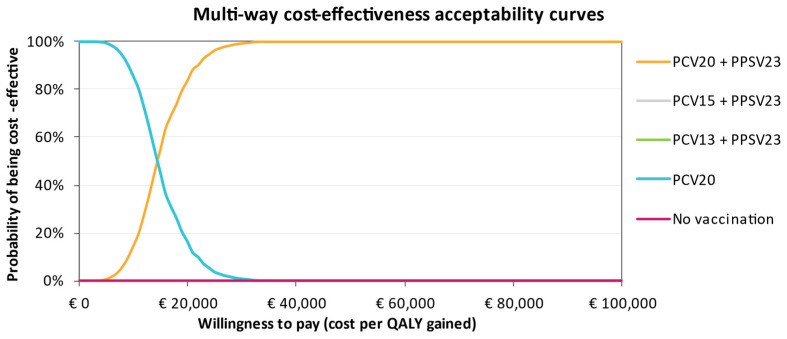
Cost-effectiveness acceptability curve of all vaccination strategies for the at-risk group. Where comparators are seemingly not visible in the CEACs, their probability of being cost-effective is 0%.

**Figure 7:**
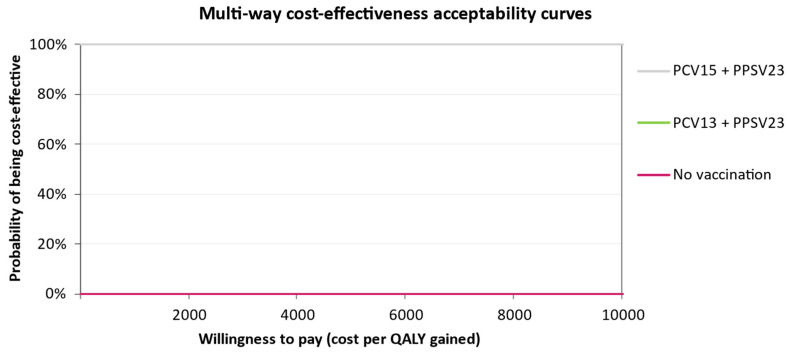
Cost-effectiveness acceptability curve of all vaccination strategies for the high-risk group. The probability of being cost-effective is 100% for PCV15 + PPSV23, and 0% for PCV13 + PPSV23.

The CEAC in Figure 5 for individuals at low risk shows that not vaccinating has the highest probability of being cost-effective at a willingness-to-pay (WTP) threshold of €40,000. As the WTP threshold increases, single and sequential vaccination with PCV20 become more likely to be cost-effective compared to No Vaccination. Vaccination with PCV20 alone is more cost-effective in comparison with sequential regimens with either PCV15 or PCV13, although the ICUR for PCV20 could still be considered above the Italian WTP threshold. In the at-risk group, PCV20 + PPSV23 and PCV20 dominate the CEAC, where the other interventions were not cost-effective, regardless of the WTP threshold (Figure 6). Conversely, in the high-risk group, PCV15 + PPSV23 is cost-effective regardless of the WTP, with a probability of 100%. This is clearly visible from the CEAC displayed in Figure 7.

### 3.4. Scenario Analyses

When applying GMT ratios to the baseline serotype-specific VE of PCV15 and PCV20, combination vaccinations PCV15 + PPSV23 and PCV20 + PPSV23 continue to dominate PCV13 + PPSV23 (Table 9). Compared with the base case serotype-specific VE, GMT-based VE affects particularly serotypes 3, 14, 22F, and 8, all of which reflect a relatively large portion of the baseline PD incidence in Italy. However, applying the GMT-based VE increases serotype-specific VE of PCV15 more than PCV20, which results in PCV15 + PPSV23 being dominant over PCV20 + PPSV23 in the low-risk population. In the at-risk population, the health gains of PCV15 + PPSV23 are more comparable to PCV20 + PPSV23’s outcomes, which is mainly due to PCV20’s greater serotype coverage, leading to a greater benefit over PCV15, as PD incidence is higher in the at-risk group. Furthermore, in this scenario, PCV20 no longer dominates PCV15 + PPSV23 in the low-risk and at-risk groups. Rather, PCV15 + PPSV23 is now more effective, albeit at ICURs of €175,909 and €91,825 for the low-risk and at-risk groups, respectively.

Scenarios assessing the effect of changes in vaccine coverage rates, price parity of PCVs, and lower PD incidence rates show similar results as in the base case (Supplementary Material, Tables S8–S13). In these analyses, PCV15 + PPSV23 remains more cost-effective compared with PCV13 + PPSV23 across all risk groups in all scenarios. Sequential vaccination with PCV20 remains more cost-effective than sequential vaccination with PCV15 in patients at low risk and at risk when the vaccine coverage rate is increased and when IPD and NBPP incidence rates are halved to reflect actual observed Italian IPD and NBPP incidence as well. When PCV15 is discounted at a rate of 5%, the base case conclusions remain unchanged for the at-risk and high-risk groups. However, for patients at low risk, PCV15 + PPSV23 becomes marginally cheaper than PCV20 + PPSV23, albeit at lower (unchanged) health gains. The results of PCV15 + PPSV23’s cost-effectiveness compared with No Vaccination are in line with the main results, where for most scenarios, the quality of life improves against increased costs in the low-risk and at-risk groups. In high-risk patients, PCV15 + PPSV23 dominates No Vaccination in the high-risk group in all scenarios but with NBPP incidence halved. The latter results in a positive ICUR of €2027 due to the additional costs of vaccination, while the benefit of vaccination is limited in this scenario.

Periods over which VE waning to 0% was modeled differed between 10 and 20 years in the literature [10]. Since the period modeled in the base case analysis falls into the longer spectrum of patterns, an alternative scenario is conducted where PCV efficacy declines over a 10-year period. Additionally, a second scenario is conducted where an inverse logarithmic pattern is modeled over the base case time periods of 20 years for PCVs and 15 years for PPSV23. Although the waning scenarios resulted in different numbers (Tables S1 and S13), both scenarios did not result in any other conclusion than can be made within the base case analysis, indicating that sequential vaccination with either PCV15 or PCV20 dominates the current vaccination regimen in Italian adults.

The scenarios combining OPA GMT ratios with a discounted price for PCV15 at 5% and increasing coverage rates equal to the individual’s VCR scenario result in comparable incremental cost-effectiveness outcomes to the individual scenarios, although PCV15 + PPSV23 shows particular improved cost-effectiveness compared with PCV20 in the at-risk population for the combined scenario of OPA GMT ratios for PCV15 and PCV20 along with an increased coverage rate of 100%. The resulting ICUR is here €26,647 per QALY gained.

## 4. Discussion

### 4.1. Base Case Findings

For the low-risk, at-risk, and high-risk groups, the results showed that sequential vaccination with PCV15 or PCV20 in combination with PPSV23 is preferred over sequential vaccination with PCV13 + PPSV23. Both dominated PCV13 + PPSV23 in all scenario analyses, having equal or lower costs, and being more effective in preventing PD cases. Both sequential vaccination and single vaccination with PCV20 dominated PCV15 + PPSV23 in the low-risk group, although the differences in effectiveness between these regimens were minor. PCV20 regimens were also dominant in the at-risk group due to a marginal difference in NBPP cases and associated costs, and there were subsequent higher QALY gains compared with PCV15 + PPSV23. This was observed in all scenarios but one, where PCV15 + PPSV23 led to lower costs when PCV15 was discounted at 5%. When compared with No Vaccination, total societal costs and QALY gains in the at-risk group were higher for PCV15 + PPSV23. With the increase in costs again predominantly being driven by vaccine acquisition and administration costs, the resulting ICUR of PCV15 + PPSV23 compared with No Vaccination was modest at €3605 per QALY gained, which was significantly below the WTP threshold of €40,000 for Italy [38]. Considering the total number of Italian adults at risk (*N* = 10,255,405), sequential regimens are of particular interest in preventing PD. In high-risk patients, vaccination with PCV15 + PPSV23 dominated No Vaccination in all but one scenario where the baseline NBPP incidence was lowered. The resulting ICUR was €2027 per QALY gained, confirming the cost-effective use of resources to immunize those at higher risk of PD disease.

### 4.2. Sensitivity and Scenario Analyses Findings

The main inputs driving the base case analysis results were those related to serotype 3-specific AMR in NBPP. Serotype 19A-specific AMR in NBPP was impactful in all three risk groups as well, for PCV20 and PCV15 in the low-risk and at-risk groups, and for PCV15 and PCV13 in the high-risk group, although to a lesser degree. These results are intuitive considering AMR is particularly high in serotypes 3 and 19A (Table 2), driving NBPP incidence and consequently NBPP associated costs up, which is exactly what is observed in the base case. Despite the larger variability of the ICUR when varying serotype 3- and 19A-specific AMR compared with the subsequent most influential parameters, sequential vaccination with PCV20 dominates sequential vaccination with PCV13 + PPSV and PCV15 + PPSV23 in all. The base case findings are therefore robust despite the margin of uncertainty surrounding serotype-specific AMR.

Similar to findings presented in earlier cost-effectiveness analyses of PCVs in the Netherlands, parameters on the baseline utilities for patients aged between 75 and 89 years, albeit for those at low-risk and at-risk instead of at high-risk, were accounted for in the top ten most influential parameters [39]. However, as was the case in the Dutch analysis, the change in ICUR was limited when varying these utilities compared with the changes seen when varying AMR parameters. This is consistent with other health–economic analyses as well, where baseline utilities did not contribute as main drivers to changes in cost-effectiveness outcomes [36,37,40]. Parameters informing vaccine effectiveness were often identified as the main drivers of model outcomes but lacked the granularity and variability incorporated in our model. 

The PSA results aligned with the results found in the main analysis. Most evidently, it showed that the dominance of PCV15 + PPSV23 over PCV13 + PPSV23 in patients at high risk is robust. A similar focus on the benefits of the introduction of PCV15 in high-risk immunosuppressed patients was recently published by Deb A. (2021) for Switzerland [14]. In this study, the proportion of subjects in immunocompetent and immunosuppressed groups was estimated at 6.7%, and 3.8%, respectively, of the total Swiss population, and immunization with PCV15 led to a favorable incremental cost-effectiveness ratio (CHF 15,616/QALY) from a societal perspective.

The stratification of surveillance data into low-risk, at-risk, and high-risk groups resulted in low estimated IPD incidence in the low-risk group, which was primarily due to its underrepresentation. Consequently, the cost-effectiveness of sequential vaccination may be adversely affected in the low-risk group compared to No Vaccination, considering the underreported PD incidence. To address this concern, baseline incidence was adjusted using TESSy rates in the analysis. Alternative scenarios were explored to evaluate the effects of the original underreported rates, demonstrating similar cost-effectiveness profiles when scaling down the upscaled NBPP and IPD incidence. A potential consequence is that the actual PD incidence—and therefore the efficacy of all vaccines—is overestimated. However, this would likely not change the order of most (cost-)effective vaccine strategies. Additionally, the adjusted national surveillance incidence, derived from 2019 data, accounts for a proportion of Italian adults who had already received prior vaccination, potentially leading to a reduction in invasive pneumococcal disease (PD) incidence. Although PD incidence increased from 2017 to 2020, it remains uncertain to what extent vaccination has decreased PD incidence. Consequently, comparing sequential vaccination to No Vaccination may exhibit a bias favoring No Vaccination. However, this bias is not anticipated to alter the conclusions presented in this analysis regarding the potential benefits of sequential vaccination for Italian adults. Unfortunately, due to the lack of empirical data on the vaccination’s reduction effect on PD incidence, we were unable to conduct scenario analyses that further quantify this effect.

In the low-risk and high-risk groups, the results of the GMT scenarios showed PCV15 + PPSV23 to be dominant over the other sequential vaccines. This differed from the base case findings. Furthermore, single vaccination with PCV20 was not dominant over PCV15 + PPSV23 anymore in the low-risk and at-risk groups. The cost-effectiveness of PCV15 + PPSV23 versus PCV20 improved further when OPA GMT ratios were combined with an increased VCR in patients at risk, whereas the ICUR dropped to €26,647 per QALY in the combined scenario compared with the OPA GMT ratios-only scenario, which resulted in an ICUR of €91,825 per QALY. These findings suggest that if real-world studies would confirm a difference in vaccine effectiveness of PCV15 and PCV20 versus PCV13, PCV15 + PPSV23 could prove a highly immunogenic and effective vaccination regime. Simultaneously, the benefits of PCV15 + PPSV23 in individuals at high risk of PD over the current strategy were clear and confirmed the main and sensitivity results. To our knowledge, this is the first cost-effectiveness analysis including both the newly approved conjugate vaccines versus PCV13-based regimens, including the sequential vaccination.

### 4.3. Analysis Strengths

The multi-state Markov model used for this cost-effectiveness analysis was based on a former version of a state-transition Markov model used to conduct multiple cost-effectiveness analyses of alternative pneumococcal vaccines and was cross-validated using the systematic literature review outcomes by Shiri et al. [41]. Additional functionalities, serotype-specific efficacy, costs of antibiotic resistance and time-dependent vaccine waning improved the current model’s flexibility. Additionally, the model allowed for the inclusion of multiple cohorts of patients, allowing for a distinction between patients with various risk profiles. When properly informed, serotype-specific efficacy allowed for a more granular distinction between vaccine-specific outcomes.

### 4.4. Comparison with Existing Literature 

The results found here for the Italian setting are consistent with those found in the analysis by Hu et al., who analyzed the health–economic outcomes of pediatric IPD associated with PCV15-specific serotypes and established that they contributed significantly to IPD-related costs and morbidity, hence establishing the need for broader serotype coverage of serotypes responsible for the disease, depending on the given context [9].

The Italian national immunization technical advisory group introduced sequential vaccination in 2013 due to the Italian-specific epidemiology of its aging population and the high prevalence of serotypes 3 and 8—which were not covered by PCV13—circulating in this population. Therefore, evaluating the cost-effectiveness of maintaining sequential regimens in such a context was a primary objective of this study. The recommendation of sequential vaccination is further supported by results following a cost-effectiveness analysis by the Centers for Disease Control and Prevention that found that sequential vaccination with PCV15 in combination with PPSV23 was both cheaper and led to improved quality of life in patients at low risk [42]. Sequential vaccination with PCV15 improved health outcomes in patients between the ages of 19 and 64 years as well, although this was at higher costs compared with the recommended strategy. Interestingly, one study evaluating the blunting of immune response of revaccination with PPSV23 as compared with sequential vaccination of PCVs following PPSV23 found that “responses to PPSV23 were comparable to those seen after initial PCV dose for shared vaccine serotypes” and reiterated that “PPSV23 vaccination of at-risk adults is essential to ensure broad protection against all 23 vaccine serotypes until PCVs will prove their effectiveness against invasive forms” [43]. This elicits interest for reevaluating the position of PPSV23 in the consideration of sequential vaccination in individuals at moderate risk when aligning with the findings presented here for those individuals with a moderate risk profile.

### 4.5. Limitations

A limitation of our study could be that herd immunity and serotype replacement, although explicitly modeled, were not captured for the Italian setting. Although with the introduction of PCV13 + PPSV23 in 2013, very high vaccination levels have been attained in Italian children (>90%), vaccine coverage rates are not standardly reported for adults in the Italian surveillance system, and they are clinically assumed to be low [44]. As a result, herd immunity or serotype replacement would have a limited effect on the model outcomes and only add another uncertainty factor. Unfortunately, data on the serotype replacement in different age groups are lacking in Italy as well due to the poor reporting in the national surveillance systems. Due to the lack of complete and robust data availability for informing herd immunity and serotype replacement, a dynamic model allowing for varying serotype incidence over time would effectively not differ from the Markov model that was introduced here. As there would be high uncertainty in the potential effects of herd immunity, a dynamic model would not provide additional insights compared with the Italian Markov model. The value of including these phenomena did not offset the potential uncertainty they would add to the model analyses, and such herd immunity and serotype replacement inputs were not populated.

The accuracy of the modeled outcomes was further limited by the uncertainty surrounding PCV15’s and PCV20’s real-world VE. Although not ideal, the assumption regarding equal VE across all PCVs is a conservative approach aiming to minimize potential bias toward PCV15 and PCV20. The scenario analyses using GMT as a proxy for VE did partly account for this uncertainty. However, inferring VE from OPA GMT ratios is an explorative methodological assumption, and its results deserve a discussion, as the alternative is to consider all new conjugate vaccines equally effective [14]. More research on the relationship between GMT and VE would be needed to support this assumption. In addition to assumptions of VE, vaccine waning is a setting that comes with uncertainty as well. The pattern and duration of waning affects incremental model outcomes substantially and introduces uncertainty, particularly around effectiveness in the absence of long-term data. An upside of the implementation of vaccine waning, as described in the section ‘Section 2.3’, however, is that it leads to intuitive results. This makes the model’s outcome straightforward for use by multiple parties.

## 5. Conclusions

Collectively, the base case and all scenarios showed that sequential vaccination with either PCV15 or PCV20 in combination with PPSV23 leads to better health outcomes compared with the currently recommended vaccination strategy and with No Vaccination. Particularly, in patients at high risk, PCV15 + PPSV23 leads to better outcomes at a lower PD burden and lower societal costs against the currently recommended strategy, emphasizing the benefits that can be gained in this population. Sequentially vaccinating patients at low risk was not cost-effective at the WTP of €40,000, where costs were high compared with the marginal health benefit these individuals gain from vaccination Additional scenarios exploring the effects of clinically derived OPA GMT ratios showed that PCV15 + PPSV23 led to lower costs and greater health gains compared with sequential vaccination with PCV13 and PCV20 in the high-risk and low-risk groups, respectively. Furthermore, our analysis showed that any vaccination alternative is beneficial and maintains the same cost-effectiveness profile when reaching the targeted 75% coverage rate, which should be the primary objective of any public health strategy in an epidemiological context such as the one well captured by the three risk groups, with the at-risk and the high-risk groups representing together the vast majority of the eligible population. The societal benefits of reaching optimal VCRs would have been even more tangible if the model incorporated herd immunity.

The model supports the continuation of the sequential strategy (PCV combined with PPSV23), with the substitution of PCV13 with PCV15 or PCV20 according to the risk profile of the target group, and the protraction of the usage of PPSV23, which is affordable and consistently associated with increased health gains. Our findings suggest that decision-makers in Italy should evaluate new pneumococcal vaccinations according to a specific epidemiological context and their immunogenic profile. Furthermore, when assessing multiple immunization options simultaneously, these options should be encompassed into a common evaluation framework.

## Figures and Tables

**Table 1:** Serotype-specific vaccine efficacy of PCVs (PCV13, PCV15, PCV20) and PPSV23 in the base case, equal across all risk groups.

Serotype	PCVs ^1^		PPSV23		
	IPD ^2,3^	NBPP ^4^	Serotype	IPD ^5^	NBPP ^6^
ST 3	26%	23%	All STs	73%	33.5%
All STs but 3	75%	45%			
NVT bucket	0%	0%	NVT bucket	0%	0%

PCV Pneumococcal conjugate vaccine; PPSV Pneumococcal polysaccharide vaccine; IPD Invasive pneumococcal disease; NBPP Non-bacteremic pneumococcal pneumonia; ST Serotype; NVT Non-vaccine type. ^1^ PCV15 and PCV20 reported IPD and NBPP vaccine efficacy assumed equal to PCV13 vaccine efficacy. ^2^ PCV IPD efficacy based on Ochoa-Gondar et al. (2014) [21]. ^3^ PCV IPD and NBPP efficacy for serotype 3 based on Pilishvil and separated out, since it is assumed to be particularly influential and relevant to this analysis [22,23].^4^ PCV NBPP efficacy based on Bonten et al. (2015) [24]. ^5^ PPSV23 efficacy against IPD based on Falkenhorst et al. (2017) [25]. ^6^ PPSV23 efficacy against NBPP based on Suzuki et al. (2017) [26].

**Table 2:** Serotype-specific AMR in IPD and NBPP for PCVs and PPSV23.

Serotype	PCVs	PPSV23
	%AMR in IPD and NBPP ^1^	
ST 3	24%	
ST 11A	2%	
ST 19A	76%	
ST 19F	14%	
ST 22F	2%	
All other STs	0%	
NVT bucket	Varying depending on the baseline serotype distribution	

PCV Pneumococcal conjugate vaccine; PPSV Pneumococcal polysaccharide vaccine; AMR Antimicrobial resistance; IPD Invasive pneumococcal disease; NBPP Non-bacteremic pneumococcal pneumonia; ST Serotype; NVT Non-vaccine type. ^1^ IPD and NBPP serotype–specific proportions due to AMR are all based on Suaya et al. [27].

**Table 3:** Vaccine acquisition and administration costs in 2021.

Vaccine Strategy	Acquisition Cost Per Dose	Administration Cost Per Dose	Reference
Any single PCV	€44.63	€6.80	Awarded tenders in Italy and OsMed cost per DDD data GPs’ single vaccine administration tariff system [33]
PPSV23 single	€19.13		
PCV + PPSV23	€63.76	€13.60	Sum of PCV and PPSV23 prices

PCV Pneumococcal conjugate vaccine; OsMed AIFA’s Medicines Utilization Monitoring Center; DDD Daily defined dose; GP General practitioner; PPSV Pneumococcal polysaccharide vaccine.

**Table 4:** Medical and indirect costs per case for IPD, NBPP, meningitis, PMS, and AMR.

Costs Per Case in Euros ^1^ (€)		
Category	Medical Costs ^2,3^	Indirect Costs ^4,5^
IPD	€8395	€433.30
NBPP (inpatient)	€4463	€826.47
NBPP (outpatient)	€333	€417.25
Meningitis	€9227	€1460.37
PMS (first and consecutive years)	€94	Not applicable
18–29 years old	Not applicable	€1604.80
30–49 years old		€1838.40
50+ years old		€2009.60
AMR in IPD	€2518.50	€129.99
AMR in NBPP	€1338.90	€247.94

IPD Invasive pneumococcal disease; NBPP Non-bacteremic pneumococcal disease; PMS Post-meningitis sequelae; AMR Antimicrobial resistance. ^1^ Most Italian cost studies multiply NHS tariffs without inflating them. This analysis, therefore, does not inflate sourced costs either for alignment purposes with the existing literature. ^2^ Medical costs for IPD, NBPP, and meningitis are sourced from Astengo et al. [35]. ^3^ Medical costs for PMS are sourced from Mennini et al. [36]. ^4^ Indirect costs for IPD, NBPP, meningitis, and AMR are sourced from Delgeize et al. [37]. ^5^ PMS-related indirect costs are based on Ansaldi et al. [34].

**Table 5:** Total number of cases of IPD, NBPP, PMS, and deaths in the low-risk, at-risk, and high-risk groups.

No. of Events	Low-Risk Age 65 (n = 387,993)					At-Risk Ages 50–100 (n = 10,255,405)					High-Risk Ages 18–100 (n = 4,552,553)		
	PCV15 + PPSV23	PCV20 + PPSV23	PCV13 + PPSV23	PCV20 (Single)	No Vaccination	PCV15 + PPSV23	PCV20 + PPSV23	PCV13 + PPSV23	PCV20 (Single)	No Vaccination	PCV15 + PPSV23	PCV13 + PPSV23	No Vaccination
IPD cases	201	195	203	199	225	47,841	47,010	48,004	47,354	49,871	41,867	42,046	44,154
Meningitis cases	40	39	41	40	45	9568	9402	9601	9471	9974	8373	8409	8831
NBPP inpatient cases	6252	6137	6285	6212	6522	1,018,625	1,007,015	1,020,915	1,011,016	1,040,928	843,429	846,735	878,903
NBPP outpatient cases	2265	2231	2275	2257	2337	1,013,362	1,003,586	1,015,291	1,007,500	1,031,464	1,451,278	1,455,284	1,490,351
PMS cases	28	27	28	28	32	6698	6581	6721	6630	6982	5861	5886	6181
AMR cases	590	590	591	597	631	128,966	129,035	129,430	133,533	128,966	143,690	143,811	151,852
PD deaths ^a^	742	14	744	740	763	208,914	206,919	209,308	207,745	212,638	224,507	225,077	229,932

IPD Invasive pneumococcal disease; NBPP Non-bacteremic pneumococcal pneumonia; PMS Post-meningitis sequelae; PCV Pneumococcal conjugate vaccine; PMS Post-meningitis sequelae; PPSV Pneumococcal polysaccharide vaccine; AMR Antimicrobial resistance; PD Pneumococcal disease; ^a^ Deaths have been aggregated over IPD, NBPP inpatients, and NBPP outpatients.

**Table 6:** Total medical and indirect costs of PD vaccination in the low-risk and at-risk groups.

Total Costs	Low-Risk Age 65 (n = 387,993)					At-Risk Ages 50–100 (n = 10,255,405)				
	PCV15 + PPSV23	PCV20 + PPSV23	PCV13 + PPSV23	PCV20 (Single)	No Vaccination	PCV15 + PPSV23	PCV20 + PPSV23	PCV13 + PPSV23	PCV20 (Single)	No Vaccination
Acquisition	€15,708,857	€15,708,857	€15,708,857	€11,255,476	€0	€153,661,857	€153,661,857	€153,661,857	€114,424,678	€0
Admin	€3,297,939	€3,297,939	€3,297,939	€1,714,928	€0	€31,381,538	€31,381,538	€31,381,538	€17,434,188	€0
IPD	€1,191,165	€1,148,479	€1,204,274	€1,177,956	€1,364,516	€290,093,224	€284,285,798	€291,229,861	€286,441,134	€305,094,047
Meningitis	€294,692	€284,132	€297,936	€291,425	€337,579	€71,768,640	€70,331,891	€72,049,842	€70,865,118	€75,479,821
NBPP inpatient	€7,114,763	€6,988,619	€7,151,113	€7,072,914	€7,410,795	€3,410,395,384	€3,368,930,653	€3,418,569,460	€3,382,338,422	€3,491,474,514
NBPP outpatient	€3,263,961	€3,194,326	€3,284,163	€3,234,499	€3,441,777	€572,977,610	€565,102,575	€574,529,698	€572,977,610	€588,752,990
PMS	€618,706	€594,093	€626,344	€610,328	€726,565	€164,259,512	€160,789,407	€164,938,481	€162,029,684	€173,360,343
AMR	€914,930	€914,504	€915,912	€925,804	€981,449	€200,985,595	€200,959,918	€201,073,996	€201,679,448	€208,402,985

PD Pneumococcal disease; PCV Pneumococcal conjugate vaccine; PPSV Pneumococcal polysaccharide vaccine; IPD Invasive pneumococcal disease; NBPP Non-bacteremic pneumococcal pneumonia; PMS Post-meningitis sequelae; AMR Antimicrobial resistance Deterministic sensitivity analysis.

**Table 7:** Total medical and indirect costs of PD vaccination in the high-risk group.

Total Costs	High-Risk Ages 18–100 (n = 4,552,553)		
	PCV15 + PPSV23	PCV13 + PPSV23	No Vaccination
Acquisition	€140,822,386	€140,822,386	€0
Admin	€29,418,165	€29,418,165	€0
IPD	€195,237,793	€196,364,290	€211,730,448
Meningitis	€48,237,798	€48,515,923	€52,308,220
NBPP inpatient	€423,097,802	€425,219,605	€447,293,205
NBPP outpatient	€3,910,848,526	€3,926,584,461	€4,081,797,384
PMS	€243,720,914	€244,535,830	€260,648,727
AMR	€124,297,937	€125,053,666	€135,573,387

PD Pneumococcal disease; PCV Pneumococcal conjugate vaccine; PPSV Pneumococcal polysaccharide vaccine; IPD Invasive pneumococcal disease; NBPP Non-bacteremic pneumococcal pneumonia; PMS Post-meningitis sequelae; AMR Antimicrobial resistance.

**Table 8:** Incremental outcomes of PCV15 + PPSV23 versus comparator regimens.

PCV15 + PPSV23 vs.	Low-Risk Age 65 (n = 387,993)				At-Risk Ages 50–100 (n = 10,255,405)				High-Risk Ages 18–100 (n = 4,552,553)	
	PCV13 + PPSV23	PCV20 + PPSV23	PCV20 (Single)	No Vaccination	PCV13 + PPSV23	PCV20 + PPSV23	PCV20 (Single)	No Vaccination	PCV13 + PPSV23	No Vaccination
Incremental costs	−€78,281	€263,505	€6,118,416	€18,185,218	−€11,630,171	€58,642,975	€92,033,528	€56,669,841	−€19,967,763	−€65,752,706
Incremental QALYs	17	−58	−23	168	1488	−7559	−5255	15,718	2778	31,811
Incremental LYs	35	−119	−43	325	4414	−22,401	−14,493	44,783	9279	98,142
ICUR	PCV15 + PPSV23 Dominant over PCV13 + PPSV23	PCV15 + PPSV23 Dominated by PCV20 + PPSV23	PCV15 + PPSV23 Dominated by PCV20	€108,542	PCV15 + PPSV23 Dominant over PCV13 + PPSV23	PCV15 + PPSV23 Dominated by PCV20 + PPSV23	PCV15 + PPSV23 Dominated by PCV20	€3605	PCV15 + PPSV23 Dominant over PCV13 + PPSV23	PCV15 + PPSV23 Dominant over No Vaccination

PCV Pneumococcal conjugate vaccine; PPSV Pneumococcal polysaccharide vaccine; QALY Quality-adjusted life year; LY Life year; ICUR Incremental cost–utility ratio.

**Table 9:** Incremental outcomes of PCV15 + PPSV23 versus comparator regimens in the OPA GMT scenarios.

PCV15 + PPSV23 vs.	Low-Risk Age 65				At-Risk Ages 50–100				High-Risk Ages 18–100	
	PCV13 + PPSV23	PCV20 + PPSV23	PCV20 (Single)	No Vaccination	PCV13 + PPSV23	PCV20 + PPSV23	PCV20 (Single)	No Vaccination	PCV13 + PPSV23	No Vaccination
**OPA GMT ratios only**										
Incremental costs	−€171,374	−€40,640	€5,842,648	€18,092,125	−€25,444,719	€9,005,298	€45,138,016	€42,855,293	−€98,877,804	−€144,662,747
Incremental QALYs	34	4	33	185	3046	−1506	492	17,276	13,308	42,340
Incremental LYs	69	4	67	359	8909	−4920	1835	49,278	42,645	131,507
ICUR	Dominant	Dominant	€175,909	€98,009	Dominant	Dominated	€91,825	€2481	Dominant	Dominant
**OPA GMT ratios + price discount**										
Incremental costs	−€734,148	-€603,414	€5,279,874	€17,529,351	−€31,165,953	€3,284,065	€39,416,782	€37,134,059	−€103,958,813	−€149,743,756
Incremental QALYs	34	4	33	185	3046	−1506	492	17,276	13,308	42,340
Incremental LYs	69	4	67	359	8909	−4920	1835	49,278	42,645	131,507
ICUR	Dominant	Dominant	€158,966	€94,960	Dominant	Dominated	€80,186	€2150	Dominant	Dominant
**OPA GMT ratios + increased VCRs**										
Incremental costs	−€197,424	−€49,374	€6,814,461	€20,948,474	−€97,905,094	€10,292,467	€181,922,875	€172,791,981	−€207,119,789	−€310,331,707
Incremental QALYs	39	5	39	214	11,641	−2662	6827	73,963	27,868	2,759,229
Incremental LYs	79	5	80	416	34,330	−10,662	21,428	211,200	89,655	282,173
ICUR	Dominant	Dominant	€173,638	€97,928	Dominant	Dominated	€26,647	€2336	Dominant	Dominant

PCV Pneumococcal conjugate vaccine; PPSV Pneumococcal polysaccharide vaccine; OPA Opsonophagocytic activity; GMT Geometric mean titer; QALY Quality-adjusted life year; LY Life year; ICUR Incremental cost–utility ratio; VCR Vaccine coverage rate.

## Data Availability

No new data was created or analyzed in this study. Data sharing is not applicable to this study.

## Supplementary Materials

The following supporting information can be downloaded at https://www.mdpi.com/article/10.3390/vaccines11071253/s1, Table S1: Input parameters NBPP- and IPD-specific incidence and mortality in low-risk patients 65 years old, Table S2: Input parameters NBPP- and IPD-specific incidence and mortality in at-risk patients 50+ years old, Table S3: Input parameters NBPP- and IPD-specific incidence and mortality in high-risk patients 18+ years old, Table S4: Serotype-specific vaccine efficacy against IPD of the newly approved vaccines as multiplied by clinically derived GMT ratios divided up into low risk/at risk and high risk, Table S5: Serotype-specific vaccine efficacy against NBPP of the newly approved vaccines as multiplied by clinically derived GMT ratios divided up into low-risk/at-risk and high-risk, Table S6: Baseline utilities per age category for each risk group, Table S7: Utility decrements per disease health state, Table S8: Incremental outcomes of PCV15 + PPSV23 versus comparator regimens in the increasing vaccine coverage rates scenarios, Table S9: Incremental outcomes of PCV15 + PPSV23 versus comparator regimens in the PCV15 discounted at 5% scenarios, Table S10: Incremental outcomes of PCV15 + PPSV23 versus comparator regimens when IPD incidence is halved, Table S11: Incremental outcomes of PCV15 + PPSV23 versus comparator regimens when NBPP incidence is halved; Table S12: Incremental outcomes of PCV15 + PPSV23 versus comparator regimens when treatment waning follows a linear decline over shortened time horizon; Table S13: Incremental outcomes of PCV15 + PPSV23 versus comparator regimens when treatment waning follows an inverse logarithmic decline; Figure S1: Scatter plot of the incremental discounted costs against incremental discounted QALYs for all treatment strategies in the low-risk group analyzed in the PSA, Figure S2: Scatter plot of the incremental discounted costs against incremental discounted QALYs for all treatment strategies in the at-risk group analyzed in the PSA, Figure S3: Scatter plot of the incremental discounted costs against incremental discounted QALYs for all treatment strategies in the high-risk group analyzed in the PSA. Refs. [45,46] are cited in the supplementary materials.
