# Supplementary material for: Cost-Effectiveness of Pneumococcal Vaccination in Adults in Italy: Comparing New Alternatives and Exploring the Role of GMT Ratios in Informing Vaccine Effectiveness
Source: Vaccines (Basel). 2023 Jul 18;11(7):1253. doi: 10.3390/vaccines11071253 (PMC10384960; doi:10.3390/vaccines11071253)
Supplement: Supplementary file 1 [file vaccines-11-01253-s001.zip › vaccines-2456991-supplementary.pdf]

vaccines

MDPI

Supplementary Material

Model inputs

Table S1. Input parameters NBPP- and IPD-specific incidence and mortality in low-risk patients 65 years old.

Age category	IPD incidence per 100.000	NBPP incidence per 100.000[1]	Case fatality rate IPD	Case fatality rate NBPP		% meningitis in IPD cases	% PMS in IPD meningitis cases	% NBPP is hospitalized
				Outpatient	Inpatient			
50 - 54	0.85	32.5	9.7%	0.5%	9.7%	20.0%	70.0%	14.0%
55 - 59	0.85	32.5	9.7%	0.5%	9.7%	20.0%	70.0%	14.0%
60 - 64	0.85	32.5	9.7%	0.5%	9.7%	20.0%	70.0%	14.0%
65 - 69	1.91	33.6	14.0%	1.3%	14.0%	20.0%	70.0%	18.0%
70 - 74	1.91	33.6	14.0%	1.3%	14.0%	20.0%	70.0%	18.0%
75 - 79	3.34	123	14.0%	4.2%	14.0%	20.0%	70.0%	23.0%
80 - 84	3.34	123	14.0%	4.2%	14.0%	20.0%	70.0%	23.0%
85 - 89	3.34	238	14.0%	10.2%	14.0%	20.0%	70.0%	30.0%
90 - 94	3.34	238	14.0%	10.2%	14.0%	20.0%	70.0%	30.0%
95 - 100	3.34	274	14.0%	10.2%	14.0%	20.0%	70.0%	50.0%

NBPP Non-bacteremic pneumococcal pneumonia; *IPD* Invasive pneumococcal disease; *PMS* Post-meningitis sequelae.[1] Incidence aggregated over inpatients and outpatients.

Table S2. Input parameters NBPP- and IPD-specific incidence and mortality in at-risk patients 50+ years old.

Age category	IPD incidence per 100.000	NBPP incidence per 100.000[1]	Case fatality rate IPD	Case fatality rate NBPP		% meningitis in IPD cases	% PMS in IPD meningitis cases	% NBPP is hospitalized
				Outpatient	Inpatient			
50 - 54	3.64	405	9.7%	0.8%	9.7%	20.0%	70.0%	25.0%
55 - 59	8.37	384	9.7%	0.8%	9.7%	20.0%	70.0%	28.0%
60 - 64	22.64	419	9.7%	1.8%	9.7%	20.0%	70.0%	28.0%
65 - 69	32.06	656	14.0%	1.8%	14.0%	20.0%	70.0%	37.0%
70 - 74	32.06	656	14.0%	1.8%	14.0%	20.0%	70.0%	37.0%
75 - 79	21.71	955	14.0%	5.1%	14.0%	20.0%	70.0%	47.0%
80 - 84	21.71	955	14.0%	5.1%	14.0%	20.0%	70.0%	47.0%
85 - 89	21.71	1903	14.0%	12.1%	14.0%	20.0%	70.0%	60.0%
90 - 94	21.71	1903	14.0%	12.1%	14.0%	20.0%	70.0%	60.0%
95 - 100	21.71	2081	14.0%	12.1%	14.0%	20.0%	70.0%	75.0%

NBPP Non-bacteremic pneumococcal pneumonia; *IPD* Invasive pneumococcal disease; *PMS* Post-meningitis sequelae. [1] Incidence aggregated over inpatients and outpatients.

Table S3. Input parameters NBPP- and IPD-specific incidence and mortality in high-risk patients 18+ years old.

Age category	IPD incidence per 100.000	NBPP incidence per 100.000[1]	Case fatality rate IPD	Case fatality rate NBPP		% meningitis in IPD cases	% PMS in IPD meningitis cases	% NBPP is hospitalized
				Outpatient	Inpatient			
50 - 54	13.76	1529	9.7%	2.3%	6.0%	20.0%	70.0%	43.0%

55 - 59	17.93	919	9.7%	2.3%	6.0%	20.0%	70.0%	43.0%
60 - 64	36.99	764	9.7%	2.3%	12.0%	20.0%	70.0%	43.0%
65 - 69	40.91	947	14.0%	2.3%	15.0%	20.0%	70.0%	55.0%
70 - 74	40.91	947	14.0%	2.3%	15.0%	20.0%	70.0%	55.0%
75 - 79	35.06	1784	14.0%	5.9%	15.0%	20.0%	70.0%	70.0%
80 - 84	35.06	1784	14.0%	5.9%	15.0%	20.0%	70.0%	70.0%
85 - 89	35.06	3669	14.0%	14.0%	15.0%	20.0%	70.0%	91.0%
90 - 94	35.06	3669	14.0%	14.0%	15.0%	20.0%	70.0%	91.0%
95 - 100	35.06	3669	14.0%	14.0%	15.0%	20.0%	70.0%	91.0%

NBPP Non-bacteremic pneumococcal pneumonia; *IPD* Invasive pneumococcal disease; *PMS* Post-meningitis sequelae. [1] Incidence aggregated over inpatients and outpatients.

Table S4. Serotype-specific vaccine efficacy against IPD of the newly approved vaccines as multiplied by clinically derived GMT ratios divided up into low risk/at risk and high risk.

Serotype	Vaccine efficacy against IPD		
	PCV15		PCV20
	Low-risk age 65 and At-risk ages 50+	High-risk ages 18+	Low-risk age 65 and At-risk ages 50+
ST 1	71%	89%	60%
ST 3	39%	42%	22%
ST 4	56%	42%	61%
ST 5	62%	91%	62%
ST 6AC	92%	85%	57%
ST 6B	95%	100%	62%
ST 7F	62%	74%	65%
ST 8	-		40%
ST 9V	66%	100%	70%
ST 10A	-		95%
ST 11A	-		95%
ST 12F	-		95%
ST 14	68%	88%	75%
ST 15BC	-		95%
ST 18C	95%	100%	64%
ST 19A	77%	86%	60%
ST 19F	80%	90%	60%
ST 22F	75%	85%	75%
ST 23F	95%	74%	62%
ST 33F	75%	85%	75%
NVT bucket	0%	0%	0%

IPD Invasive pneumococcal disease; *GMT* Geometric mean titer; *PCV* Pneumococcal conjugate vaccine; *ST* Serotype; *NVT* Non-vaccine type.

Table S5. Serotype-specific vaccine efficacy against NBPP of the newly approved vaccines as multiplied by clinically derived GMT ratios divided up into low-risk/at-risk and high-risk.

Serotype	Vaccine efficacy against NBPP				
	PCV15			PCV20	
	Low-risk Age 65	At-risk Ages 50+	High-risk Ages 18+	Low-risk Age 65	At-risk Ages 50+
ST 1	43%		54%	36%	

ST 3	35%	37%	20%
ST 4	33%	25%	36%
ST 5	37%	55%	37%
ST 6AC	55%	51%	34%
ST 6B	72%	60%	37%
ST 7F	37%	45%	39%
ST 8	-		18%
ST 9V	40%	60%	42%
ST 10A	-		62%
ST 11A	-		59%
ST 12F	-		50%
ST 14	41%	53%	45%
ST 15BC	-		95%
ST 18C	69%	87%	38%
ST 19A	46%	51%	36%
ST 19F	48%	54%	36%
ST 22F	45%	53%	45%
ST 23F	60%	44%	37%
ST 33F	45%	53%	45%
NVT bucket	0%	0%	0%

NBPP Non-bacteremic pneumococcal pneumonia; *GMT* Geometric mean titer; *PCV* Pneumococcal conjugate vaccine; *ST* Serotype; *NVT* Non-vaccine type.

Table S6. Baseline utilities per age category for each risk group.

Age category	Baseline utility	
	Low-risk	At-risk & High-risk
18-24	0.92	0.72
25-29	0.91	0.72
30-34	0.91	0.72
35-39	0.84	0.72
40-44	0.84	0.72
45-49	0.82	0.72
50-54	0.82	0.72
55-59	0.82	0.69
60-64	0.82	0.63
65-69	0.82	0.57
70-74	0.82	0.54
75-79	0.81	0.52
80-84	0.81	0.51
85-89	0.81	0.51
90-94	0.81	0.51
95-100	0.81	0.51

Utilities retrieved from Sisk et al. 2003 [43].

Table S7. Utility decrements per disease health state.

Category	Daily decrement (value)	Decrement days
IPD	0.0709	5.40

NBPP outpatients	0.0045	10.30
NBPP inpatients	0.0709	5.20
Meningitis	0.0709	18.20
AMR in IPD	0	6.40 (ages 18-49)<br>7.00 (ages 50-64)<br>7.10 (ages 65+)
AMR in NBPP	0	
PMS[1]	0.690	NA

IPD Invasive pneumococcal disease; *NBPP* Non-bacteremic pneumococcal disease; *AMR* Antimicrobial resistance; *PMS* Post-meningitis sequelae. [1]As opposed to utility decrements, regular utilities are reported for PMS. [2] The number of decrement days and decrement utility values were obtained from Stoecker et al, Mangen et al, and des Portes [14,44,45].

Scenario analyses results

Table S8. Incremental outcomes of PCV15+PPSV23 versus comparator regimens in the increasing vaccine coverage rates scenarios.

PCV15 + PPSV23 vs	Low-risk Age 65			At-risk Ages 50-100			High-risk Ages 18-100	
	PCV13 + PPSV23	PCV20 + PPSV23	No Vaccination	PCV13 + PPSV23	PCV20 + PPSV23	No Vaccination	PCV13 + PPSV23	No Vaccination
**Incremental costs**	-89,843	302,092	21,056,054	-31,767,718	160,687,794	171,463,670	-29,244,886	-101,132,999
**Incremental QALYs**	20	-66	194	4,050	-20,640	50,342	4,076	48,486
**Incremental LYs**	40	-136	377	12,174	-61,964	143,544	13,697	149,676
**ICUR**	PCV15 + PPSV23 Dominant over PCV13 + PPSV23	PCV15 + PPSV23 Dominated by PCV20 + PPSV23	€ 108,418	PCV15 + PPSV23 Dominant over PCV13 + PPSV23	PCV15 + PPSV23 Dominated by PCV20 + PPSV23	€ 3,406	PCV15 + PPSV23 Dominant over PCV13 + PPSV23	PCV15 + PPSV23 Dominant over No Vaccination

PCV Pneumococcal conjugate vaccine; *PPSV* Polysaccharide vaccine; *QALY* Quality-adjusted life year; *LY* Life year; *ICUR* Incremental cost-utility ratio.

Table S9. Incremental outcomes of PCV15+PPSV23 versus comparator regimens in the PCV15 discounted at 5% scenarios.

PCV15 + PPSV23 vs	Low-risk Age 65			At-risk Ages 50-100			High-risk Ages 18-100	
	PCV13 + PPSV23	PCV20 + PPSV23	No Vaccination	PCV13 + PPSV23	PCV20 + PPSV23	No Vaccination	PCV13 + PPSV23	No Vaccination
**Incremental costs (€)**	-641,055	-299,269	17,622,444	-7,351,405	52,921,741	50,948,607	-25,048,771	-70,833,715
**Incremental QALYs**	17	-58	168	1,488	-7,559	15,718	2,778	31,811
**Incremental LYs**	35	-119	325	4,414	-22,401	44,783	9,279	98,142
**ICUR**	PCV15	€ 5,168	€ 105,183	PCV15 +	PCV15 +	€ 3,241	PCV15 +	PCV15 +

	+ PPSV23 Dominant over PCV13 + PPSV23			PPSV23 Dominant over PCV13 + PPSV23	PPSV23 Dominated by PCV20 + PPSV23		PPSV23 Dominant over PCV13 + PPSV23	PPSV23 Dominant over No Vaccination

PCV Pneumococcal conjugate vaccine; *PPSV* Polysaccharide vaccine; *QALY* Quality-adjusted life year; *LY* Life year; *ICUR* Incremental cost-utility ratio.

Table S10. Incremental outcomes of PCV15+PPSV23 versus comparator regimens when IPD incidence is halved.

PCV15 + PPSV23 vs	Low-risk Age 65			At-risk Ages 50-100			High-risk Ages 18-100	
	PCV13 + PPSV23	PCV20 + PPSV23	No Vaccination	PCV13 + PPSV23	PCV20 + PPSV23	No Vaccination	PCV13 + PPSV23	No Vaccination
**Incremental costs (€)**	-67,881	229,868	18,328,491	-10,728,877	54,048,471	68,840,190	-19,001,412	-51,840,670
**Incremental QALYs**	15	-52	141	1,383	-7,022	14,301	2,653	29,910
**Incremental LYs**	31	-108	276	4,096	-20,778	40,670	8,849	92,252
**ICUR**	PCV15 + PPSV23 Dominant over PCV13 + PPSV23	PCV15 + PPSV23 Dominated by PCV20 + PPSV23	€130,123	PCV15 + PPSV23 Dominant over PCV13 + PPSV23	PCV15 + PPSV23 Dominated by PCV20 + PPSV23	€4,813	PCV15 + PPSV23 Dominant over PCV13 + PPSV23	PCV15 + PPSV23 Dominant over No Vaccination

PCV Pneumococcal conjugate vaccine; *PPSV* Polysaccharide vaccine; *IPD* Invasive pneumococcal disease; *QALY* Quality-adjusted life year; *LY* Life year; *ICUR* Incremental cost-utility ratio.

Table S11. Incremental outcomes of PCV15+PPSV23 versus comparator regimens when NBPP incidence is halved.

PCV15 + PPSV23 vs	Low-risk Age 65			At-risk Ages 50-100			High-risk Ages 18-100	
	PCV13 + PPSV23	PCV20 + PPSV23	No Vaccination	PCV13 + PPSV23	PCV20 + PPSV23	No Vaccination	PCV13 + PPSV23	No Vaccination
**Incremental costs (€)**	-49,576	165,508	18,452,431	-6,771,536	34,184,828	108,109,467	-11,123,725	36,460,727
**Incremental QALYs**	10	-35	111	854	-4,341	9,329	1,531	17,986
**Incremental LYs**	21	-71	212	2,543	-12,914	26,687	5,146	55,701
**ICUR**	PCV15 + PPSV23 Dominant over PCV13 + PPSV23	PCV15 + PPSV23 Dominated by PCV20 + PPSV23	€ 166,977	PCV15 + PPSV23 Dominant over PCV13 +	PCV15 + PPSV23 Dominated by PCV20 + PPSV23	€ 11,588	PCV15 + PPSV23 Dominant over PCV13 +	€ 2,027

	PPSV23	PPSV23

PCV Pneumococcal conjugate vaccine; *PPSV* Polysaccharide vaccine; *NBPP* Non-bacteremic pneumococcal pneumonia; *QALY* Quality-adjusted life year; *LY* Life year; *ICUR* Incremental cost-utility ratio

Table S12. Incremental outcomes of PCV15+PPSV23 versus comparator regimens when treatment waning follows a linear decline over shortened time horizon.

PCV15 + PPSV23 vs	Low-risk Age 65			At-risk Ages 50-100			High-risk Ages 18-100	
	PCV13 + PPSV23	PCV20 + PPSV23	No Vaccination	PCV13 + PPSV23	PCV20 + PPSV23	No Vaccination	PCV13 + PPSV23	No Vaccination
**Incremental costs (€)**	−€92,934	−€121,715	€5,962,367	€18,499,257	−€27,054,533	−€32,381,340	€37,669,733	€79,107,893
**Incremental QALYs**	21	28	15	108	3,629	4339	1966	13,556
**Incremental LYs**	37	49	27	191	9182	10,977	5470	35,240
**ICUR**	Dominant	Dominant	€406,706	€171,855	Dominant	Dominant	€19,157	€5836

PCV Pneumococcal conjugate vaccine; *PPSV* Polysaccharide vaccine; *IPD* Invasive pneumococcal disease; *QALY* Quality-adjusted life year; *LY* Life year; *ICUR* Incremental cost-utility ratio.

Table S13. Incremental outcomes of PCV15+PPSV23 versus comparator regimens when treatment waning follows an inverse logarithmic decline.

PCV15 + PPSV23 vs	Low-risk Age 65			At-risk Ages 50-100			High-risk Ages 18-100	
	PCV13 + PPSV23	PCV20 + PPSV23	No Vaccination	PCV13 + PPSV23	PCV20 + PPSV23	No Vaccination	PCV13 + PPSV23	No Vaccination
**Incremental costs (€)**	−€163,809	−€212,927	€5,920,722	€18,283,877	−€47,923,318	−€57,420,471	€38,185,579	€30,299,165
**Incremental QALYs**	38	49	25	154	6301	7542	1879	19,569
**Incremental LYs**	71	92	50	284	17,447	20,884	5593	53,519
**ICUR**	Dominant	Dominant	€235,762	€119,071	Dominant	Dominant	€20,323	€1548

PCV Pneumococcal conjugate vaccine; *PPSV* Polysaccharide vaccine; *IPD* Invasive pneumococcal disease; *QALY* Quality-adjusted life year; *LY* Life year; *ICUR* Incremental cost-utility ratio.

Probabilistic sensitivity analysis results.

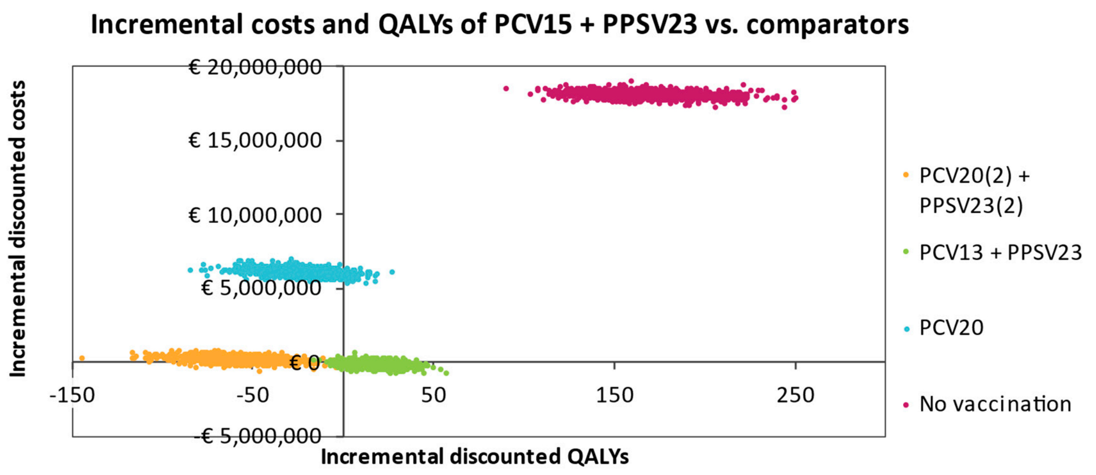

Figure S1. Scatter plot of the incremental discounted costs against incremental discounted QALYs for all treatment strategies in the low-risk group analyzed in the PSA.

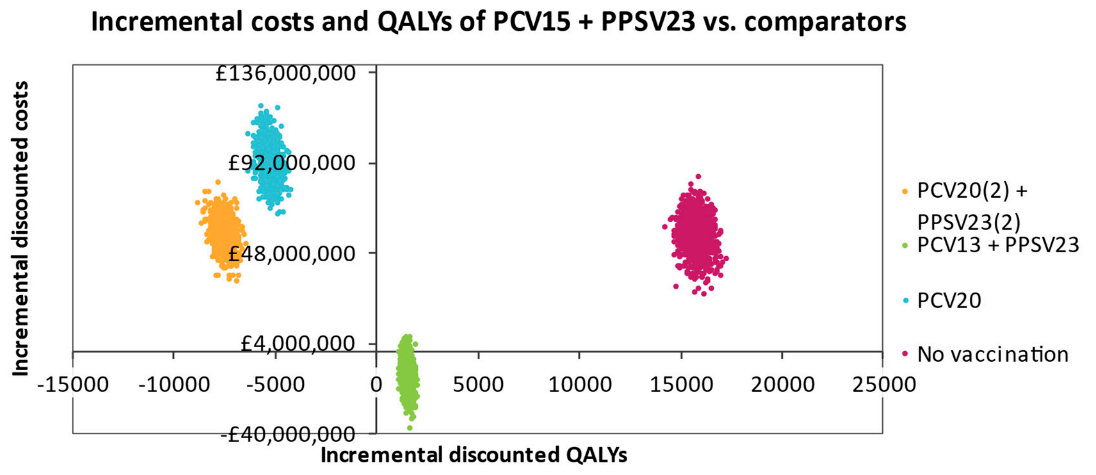

Figure S2. Scatter plot of the incremental discounted costs against incremental discounted QALYs for all treatment strategies in the at-risk group analyzed in the PSA.

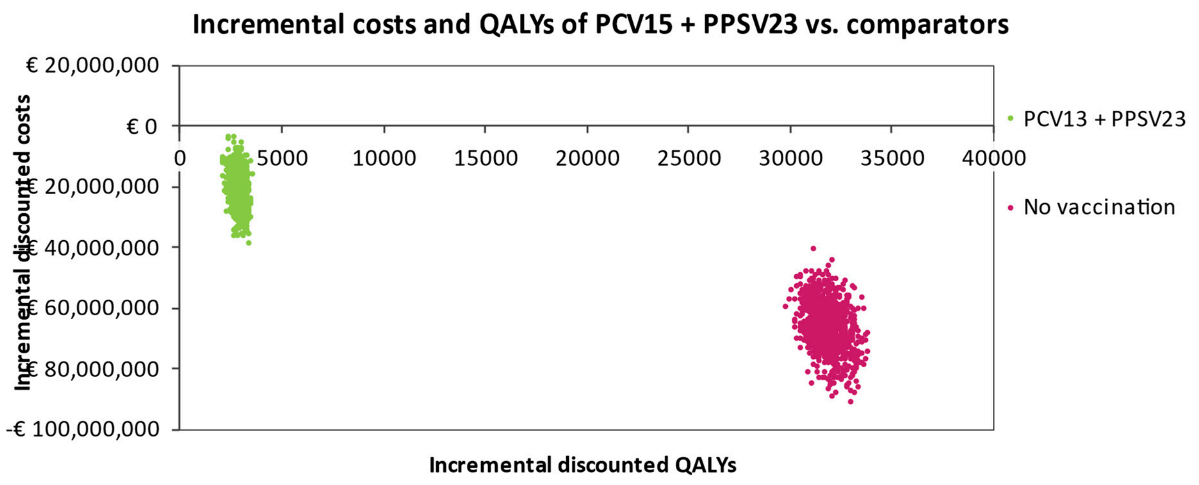

Figure S3. Scatter plot of the incremental discounted costs against incremental discounted QALYs for all treatment strategies in the high-risk group analyzed in the PSA.
